# Vaccine coverage within the first year of life and associated factors with incomplete immunization in a Brazilian birth cohort

**DOI:** 10.1186/s13690-020-00403-4

**Published:** 2020-04-08

**Authors:** Romina Buffarini, Fernando C. Barros, Mariângela F. Silveira

**Affiliations:** 1 Federal University of Pelotas, Marechal Deodoro 1160, 3rd floor, Pelotas, RS 96020-220 Brazil; 2 Catholic University of Pelotas, Gonçalves Chaves 373, Pelotas, RS 96015-560 Brazil

**Keywords:** Children, Immunization coverage, Associated factors, Cohort studies

## Abstract

**Background:**

Immunization has been held as a major achievement for global health, however, still exist many children who are not completely vaccinated. Knowledge about factors contributing to the incomplete immunization is important to develop effective strategies and interventions to achieve universal coverage to control or eradicate vaccine-preventable diseases. This study aimed to describe immunization coverage within the first year of life and associated factors with incompleteness among participants of the 2015 Pelotas Birth Cohort, Brazil.

**Methods:**

Cross-sectional analyses were performed using data from a population-based cohort. Information on vaccination status was collected from immunization cards and verbal mother’s reports from 4014 children aged 24 months. Coverage was described for each vaccine, for the basic and the complete national schedule. Incomplete vaccination was defined as failure to receive the recommended number of doses by the National Immunization Program in 2015. Bivariate and multivariate Poisson regressions with robust variance were conducted to identify factors associated with incompleteness.

**Results:**

Vaccine coverage of individual vaccines varied from 81% (Hepatitis A) to 97% (BCG). Full immunization coverage were 77% (95% CI: 75.8; 78.4) and 66.1% (95% CI: 64.6; 67.5), for basic and expanded indicators, relatively. Multivariate analyses showed that factors associated with incompleteness (for both basic and expanded coverage) were high family income, high parity, low number of prenatal consultations, not have done the tetanus toxoid, reduced diphtheria toxoid, and acellular pertussis (Tdap) vaccine during pregnancy, not have breastfeed the child until at age 12 months and not have used public health care services for child’s vaccination.

**Conclusion:**

Findings of our study show the need to develop and implement policies and programs to achieve complete immunization during the first year of life. Some strategies would include an increase in the primary health care services networks, improving their quality and access, the dissemination of scientific-based evidence about the benefits of vaccination, through communication campaigns and guidance provided by health professionals, especially those responsible for prenatal care.

## Background

Vaccines were one of the greatest public health achievements in the twentieth century, being the safest and most cost-effective intervention to control infectious diseases [1, 2]. However, still exist a high number of children with incomplete vaccination coverage, which represents a risk to the resurgence of diseases that are under control and to the reintroduction of those already eliminated [3].

In Brazil, immunization is mainly public and the vaccines are provided free of charge for the whole population through the Unified Health System (SUS, Sistema Único de Saúde) [4]. The National Program of Immunizations (PNI, Programa Nacional de Inmunizações) was first launched in 1973 and its schedule managed to eliminate four diseases (smallpox, poliomyelitis, measles andmaternal and neonatal tetanus) [5]. New vaccines were later included to control infections by *Haemophilus influenzae* type b, rotavirus and pneumococcal, making the vaccination schemes more complex [5, 6]. Over the last four decades, Brazil has made a tremendous progress in improving levels of vaccination coverage [6], however, estimates from the National Immunization System showed that coverage with key vaccines has decreased from 2014 onwards [7, 8]. A previous study from Pelotas analyzed trends in immunization coverage over a 33-year period and observed a major increase in coverage from 1980 to 1993, followed by a slightly decrease from 1993 to 2004, and further declines from 2004 to 2015 [9].

Several studies have revealed lower parental education [10–12], lower income [10, 13, 14], mother’s black skin color [10, 15], extremes of mother’s age, higher parity [16] and low number of prenatal visits [12] as barriers to complete immunization. More recent findings showed lower vaccination coverage in higher socioeconomic strata [9, 17, 18]. Increasing immunization coverage is necessary to achieve control or eradicate vaccine-preventable diseases [19]. Therefore, knowledge concerning factors associated with incompleteness are very important for decision making at the different management levels (policy makers, program managers, health workers).

In this study we aimed to describe immunization coverage of individual vaccines and two indicators of complete coverage (basic and expanded) according to the National Childhood Immunization Schedule for the first year of life, and to assess the factors associated to incompleteness for both coverage indicators. We hypothesized that incomplete vaccination was greater among children belonging to the poorest families, whose mothers had low formal education, less than 20 years of age, black skin color and high parity.

## Methods

### Study design and population

Pelotas is a Southern Brazilian city with nearly 340,000 inhabitants. In 2015, all hospital-delivered newborns (about 99% of all births) residents in the urban area of the city were eligible for the study; information was obtained on 4275 out of 4333 live births. Since then, the participants have been sought at different moments. On each occasion, pre-tested standardized questionnaires were applied by trained interviewers to the mothers or other guardian. Further details on the methodology have been published elsewhere [20].

This study included data from perinatal and 24-months follow-ups, in which all cohort members were evaluated. The visit took place at the university research clinic and response rates were 98.7 and 95.2%, respectively [20].

### Ethics

The study protocol was approved by the School of Physical Education Ethics Committee at the Federal University of Pelotas (CAAE registration number: 26746414.5.0000.5313) and written informed consent was obtained from parents or guardians in each visit.

### Outcome

Information on immunization was collected from vaccination cards shown to the interviewers and from mothers’ verbal reports. If the cards were available, the interviewer took a picture of the card. If the card there was not available or there was no record of the vaccination, then the mothers reported the total doses of the vaccines given to her child. When mothers responded with “don’t know” the child was considered as “not vaccinated”, since such response reflects a negative response regarding immunization. For all vaccines assessed, the administration was scheduled for the first year of life (Additional file 1). However, to address gaps in vaccination information and to maintain comparability among other Pelotas birth cohorts [9], data on immunization relied on the 24-month follow-up.

A child was classified as having coverage on a particular vaccine if it had received the total number of doses for the corresponding vaccine scheme, e.g. three doses for the pentavalent (diphtheria, tetanus, pertussis, haemophilus B and hepatitis B) vaccine. The child’s vaccination status was categorized as full immunization and incomplete immunization. Full immunization was defined in two ways. First, basic full immunization was defined as a child who has received the recommended routine immunizations, namely, one dose of bacille Calmette-Guérin (BCG), three doses of pentavalent vaccine, three doses of polio vaccine (oral polio vaccine -OPV−/ inactivated polio vaccine -IPV-), and one dose of measles vaccine (*measles*-mumps-rubella *vaccine -MMR-).* Second, expanded full immunization was defined as a child in receipt of the complete scheduled vaccine doses of the National Childhood Immunization Schedule of the Brazilian Ministry of Health. *F*ive extra vaccines were added to the basic full immunization indicator, i.e. three doses of 10-valent pneumococcal conjugate vaccine, two doses of B C meningococcal vaccine, two doses of rotavirus and one dose of hepatitis A.

The outcomes variables of this study were basic and expanded incomplete immunization, defined as failure to be fully immunized for the basic and expanded indicators, respectively.

### Explanatory variables

Family income, maternal age, skin colour and education, parity, number of prenatal consultations, administration of tetanus toxoid, reduced diphtheria toxoid, and acellular pertussis (Tdap) vaccine during pregnancy, breastfeeding status at age 12 months and vaccination using the public health care services (Brazilian Health System) were included as explanatory variables. Family income during the month preceding birth, collected as a continuous variable, was obtained by summing the monthly wages of all household members and then split into quintiles. Mother’s age in complete years was classified in < 20, 20 to 35, and >  35. Maternal skin colour was self-reported; three groups (black, brown and white) were coded according to the classification adopted by the Brazilian Census Bureau [21]. Maternal schooling was gathered as continuous and then categorized into four groups (0 to 4, 5 to 8, 9 to 11 and 12 or more complete years of formal education). Parity, collected as total number of live children ever born, was grouped into three categories: 1, 2 to 3, 4 or more. Number of prenatal consultations, extracted from prenatal cards, was categorized as 0 to 5 and 6 or more, according to the Brazilian Ministry of Health [22]. Administration of Tdap during pregnancy, breastfeeding at age 12 months and vaccination using the public services were classified as yes/no.

The explanatory variables were classified into three hierarchical groups, based on a theoretical model of the factors associated with incomplete childhood immunization in Brazil [10, 13, 14, 17, 18]. The more distal level included socioeconomic and demographic variables (maternal age, education, skin colour and family income), the intermediate level considered maternal variables (parity, prenatal consultations and Tdap vaccine during pregnancy); and the proximal level considered variables related to the child (breastfeeding and vaccination using the public health care services).

### Statistical analyses

Full immunization coverage was described by percentages (%) and 95% CI. Simple and multivariate Poisson regression with robust variance were used to investigate the factors associated with incomplete immunization (basic and expanded), prevalence ratios (PR) and 95% CI were estimated.

Multivariate analyses were performed considering the hierarchical analytical model. For each level, we simultaneously introduced variables from the same level, in addition to those from previous levels variables with *p*-value less than 0.20 (kept in the model as possible confounders). The estimates for each variable corresponded at the respective hierarchical level in the model. All the analyses were carried out in STATA 15.1 (StataCorp, College Station, USA).

## Results

Characteristics of the sample are shown in Additional file 2. About 70% of the mothers had at least 9 years of formal education, had 20 to 35 years of age and had white skin color. Half of mothers had 1 child and did the Tdap vaccine during pregnancy. Most of the mothers had 6 or more prenatal consultations (87%) and used public health care services for vaccination (89%). More than a half of the assessed children were nor breastfeeding at age 12 months (59%).

The hepatitis A vaccine showed the lowest coverage (81%), followed by rotavirus (83%), polio (84%) and 10-valent pneumococcal (85%). In contrast, the BCG vaccine showed the highest coverage (97%). Concerning full immunization indicators, the basic coverage was 77%, while the expanded coverage was 66% (23 and 34% of incomplete vaccination, respectively) (Table 1).

**Table 1:** Immunization coverage (%) within the first year of life in the 2015 Pelotas Birth Cohort

Immunization coverage	2015 Pelotas Birth Cohort	
	%	95% CI
BCG^a^ (1 dose)	96.9	96.3; 97.4
POLIO (3 doses)	84.1	83.0; 85.2
PENTA^b^ (3 doses)	92.9	92.1; 93.7
MMR^c^ (1 dose)	85.9	84.8; 86.9
HEPATITIS B (1 dose)	90.6	89.7; 99.5
10-VALENT PNEUMOCCOCAL (3 doses)	84.5	83.3; 85.5
C MENINGOCCOCAL (2 doses)	88.0	87.0; 89.0
ROTAVIRUS (2 doses)	83.0	81.8; 84.1
HEPATITIS A (1 dose)	81.0	79.8; 82.2
Full immunization basic	77.2	75.8; 78.4
Full immunization expanded	66.1	64.6; 67.5

^a^*BCG* Bacille Calmette-Guérin (against tuberculosis), ^b^*PENTA* Diphtheria-tetanus-pertussis, Hib and Hepatitis Bm ^c^*MMR* Measles-mumps-rubella

Missing are treated as 0 (not vaccinated)

Basic full immunization was defined as having one dose of BCG, one dose of MMR, three doses of polio vaccine, and three doses of pentavalent vaccine.

Expanded full immunization was defined as have reached the complete scheduled vaccine doses of national immunization program by age 12 months (basic + one dose of hepatitis B, one dose of hepatitis A, three doses of 10-valent pneumococcal, two doses of rotavirus and two doses of C meningococcal)

### Basic incomplete vaccination

Unadjusted analyses showed that basic incomplete immunization was higher in children belonging to the poorest and highest quintiles of family income, whose mothers had more than 35 years of age, 3 or more children, less than 6 prenatal consultations and had not done Tdap vaccine during pregnancy,children not breastfed at 12 months of age and had not used the public health care services for vaccination. In contrast, children whose mothers had 9 to 11 years of formal schooling were more likely to have complete vaccination compared with those children of mothers with the highest level of formal education (12 or more) **(**Table 2**).**

**Table 2:** Associated factors to incomplete basic immunization coverage by age 12 month. 2015 Pelotas Birth Cohort

	Incomplete basic immunisation^a^			
	Unadjusted		Adjusted	
	%	95% IC	%	95% IC
**Maternal education (years)**	< 0.001		0.012	
0 to 4	1.01	0.83; 1.23	1.17	0.93; 1.47
5 to 8	0.83	0.72; 0.96	0.98	0.82; 1.17
9 to 11	0.71	0.62; 0.82	0.84	0.72; 0.98
12 or more	1 (ref)	–	1 (ref)	–
**Family income (quintiles)**	< 0.001		< 0.001	
Q1 (poorest)	1 (ref)	–	1 (ref)	–
Q2	0.86	0.71; 1.03	0.89	0.74; 1.07
Q3	0.79	0.66; 0.96	0.83	0.68; 1.01
Q4	0.81	0.67; 0.98	0.84	0.69; 1.03
Q5 (richest)	1.29	1.09; 1.51	1.26	1.03; 1.54
**Maternal age (years)**	0.012		0.092	
< 20	1 (ref)	–	1 (ref)	–
20–35	1.13	0.65; 1.35	1.22	0.93; 1.36
> 35	1.38	1.11; 1.72	1.29	1.02; 1.63
**Maternal skin color**	0.702		0.91	
White	1 (ref)	–	1 (ref)	–
Brown	0.93	0.78; 1.11	0.96	0.81; 1.15
Black	0.97	0.82; 1.14	1.01	0.85; 1.19
**Parity**	< 0.001		< 0.001	
1 child	1 (ref)	–	1 (ref)	–
2 children	1.31	1.15; 1.49	1.37	1.18; 1.58
3 children or more	1.6	1.39; 1.84	1.73	1.46; 2.07
**Number of prenatal consultations**	< 0.001		< 0.001	
0 to 5	1.51	1.31; 1.74	1.57	1.35; 1.84
6 or more	1 (ref)	–	1 (ref)	–
**Tdap**^b^**vaccine during pregnancy**	0.004		0.025	
No	1.19	1.05; 1.34	1.15	1.02; 1.30
Yes	1 (ref)	–	1 (ref)	–
**Breastfeeding status at 12 months**	0.005		0.001	
No	1.2	1.05; 1.36	1.25	1.09; 1.42
Yes	1 (ref)	–	1 (ref)	–
**Use of public health care services**	< 0.001		0.006	
No	1.39	1.19; 1.63	1.28	1.07; 1.52
Yes	1 (ref)	–	1 (ref)	–

^a^Incomplete immunization defined as not have reached one dose of BCG, one dose of MMR, three doses of polio vaccine, and three doses of pentavalent vaccine

^b^Tdap tetanus toxoid, reduced difteria toxoid and acellular pertussis

With exception of maternal age, factors associated with basic incompleteness in crude analyses remained in the adjusted model. Children whose mothers had 9 to 11 years of formal schooling had 16% less probability of incompleteness, comparing with those whose mothers had the highest level of education (12 or more years) (PR = 0.84; 95%CI: 0.72; 0.98). Children whose families belonged to the richest quintile of income had 1.26 times more risk of having incomplete vaccination compared with those whose families were the poorest (PR = 1.26; 95%CI: 1.36; 1.94). Basic incomplete vaccination was higher with increasing parity (PR = 1.37; 95%CI: 1.18; 1.58 and PR = 1.73; 95%CI: 1.46; 2.07, having 2 children and 3 children or more compared with having 1 child, respectively). Children whose mothers had 0 to 5 prenatal consultations (PR = 1.15; 95%CI: 1.02; 1.30) and who had not Tdap vaccine during pregnancy (PR = 1.57 95%CI: 1.35; 1.84) had higher risk of incompleteness and compared with those whose mother had 6 or more prenatal consultations and had done the Tdap vaccine during pregnancy, respectively. Incompleteness was also more frequent in children who were not breastfed at age 12 months (PR = 1.25; 95%CI: 1.09; 1.42) and were not vaccinated using the public health care system (PR = 1.28; 95%CI: 1.07; 1.52) **(**Table 2**).**

### Expanded incomplete vaccination

Factors associated with expanded incompleteness resembled those associated with the basic incomplete vaccination. After controlling for confounding through multivariate model, expanded incompleteness was found to increase with a higher income of the children’s family (PR = 1.15; 95%CI: 1.00; 1.34, highest quintile in comparison with the lowest quintile). Increasing parity was also shown as a risk factor for expanded incomplete vaccination (PR = 1.23; 95%CI: 1.11; 1.37 and PR = 1.52; 95%CI: 1.35; 1.71, having 2 children and 3 children or more compared with having 1 child, respectively). Children whose mothers had less than 6 prenatal consultations and did not Tdap vaccine during pregnancy were 1.39 and 1.18 times respectively more likely to be incompletely vaccinated compared to those whose mothers had the recommended number of prenatal consultations and did the Tdap vaccine during pregnancy. Children who were not breastfed at age 12 months and children who were not vaccinated using the public services had around 23% higher risk of being incompletely vaccinated than those who were still breastfeeding at 12 months of age and were vaccinated using the public health care system, respectively (PR = 1.24; 95%CI: 1.12; 1.37 and PR = 1.22; 95%CI: 1.06; 1.39) **(**Table 3**).**

**Table 3:** Associated factors to incomplete expanded immunization coverage by age 12 month. 2015 Pelotas Birth Cohort

	Incomplete expanded immunisation^a^			
	Unadjusted		Adjusted	
	%	95% IC	%	95% IC
**Maternal education (years)**	0.001		0.024	
0 to 4	1.05	0.90; 1.22	1.14	0.95; 1.36
5 to 8	0.98	0.88; 1.09	1.09	0.95; 1.25
9 to 11	0.82	0.74; 0.92	0.92	0.82; 1.04
12 or more	1 (ref)	–	1 (ref)	–
**Family income (quintiles)**	< 0.001		< 0.001	
Q1 (poorest)	1 (ref)	–	1 (ref)	–
Q2	0.86	0.75; 0.98	0.88	0.77; 1.00
Q3	0.78	0.68; 0.90	0.82	0.71; 0.95
Q4	0.80	0.70; 0.93	0.85	0.73; 0.99
Q5 (richest)	1.10	0.97; 1.24	1.15	1.01; 1.34
**Maternal age (years)**	0.247		0.230	
< 20	1 (ref)	–	1 (ref)	–
20–35	1.06	0.94; 1.21	1.11	0.97; 1.28
> 35	1.16	0.98; 1.37	1.16	0.97; 1.39
**Maternal skin color**	0.88		0.825	
White	1 (ref)	–	1 (ref)	–
Brown	1.03	0.91; 1.17	1.04	0.91; 1.18
Black	1.02	0.90; 1.15	1.02	0.90; 1.16
**Parity**	< 0.001		< 0.001	
1 child	1 (ref)	–	1 (ref)	–
2 children	1.21	1.09; 1.34	1.23	1.11; 1.37
3 children or more	1.52	1.36; 1.68	1.52	1.35; 1.71
**Number of prenatal consultations**	< 0.001		< 0.001	
0 to 5	1.42	1.27; 1.58	1.39	1.24; 1.56
6 or more	1 (ref)	–	1 (ref)	–
**Tdap**^b^**vaccine during pregnancy**	< 0.001		< 0.001	
No	1.22	1.12; 1.33	1.18	1.07; 1.29
Yes	1 (ref)	–	1 (ref)	–
**Breastfedding status at 12 months**	< 0.001		< 0.001	
No	1.21	1.09; 1.33	1.24	1.12; 1.37
Yes	1 (ref)	–	1 (ref)	–
**Use of public health care services**	< 0.001		< 0.001	
No	1.25	1.10; 1.41	1.22	1.06; 1.39
Yes	1 (ref)	–		–

^a^Incomplete immunization defined as not have reached the complete scheduled vaccine doses of national immunization program for the first year of life

^b^Tdap: tetanus toxoid, reduced difteria toxoid and acellular pertussis

## Discussion

In a Brazilian population-based birth cohort, we found individual vaccines coverage scheduled for the first year of life ranging from 81% (Hepatitis A) to 97% (BCG). Only BCG vaccine reached the target above 90% established by the National Program of Immunization, followed by pentavalent vaccine, with two percentage points left to reach the goal of 95% [6]. In general, our results are consistent with estimates from year 2015 for the state of Rio Grande do Sul -where the city of Pelotas is located-, with similar coverage for BCG, Hepatitis B, pentavalent and MMR (around 98, 91, 92 and 86%, respectively) [23].

Regarding full immunization, about 77 and 66% of the assessed children received the basic and expanded complete vaccination schemes, respectively. The expanded full immunization coverage was lower than the basic indicator. This was expected, as the expanded vaccine schedule in 2015 was more complex and required several contacts with the health services to deliver the doses of nine vaccines during the first year of the child’s life (Additional file 1), thus, reducing the coverage rates [24]. However, the factors which contributed to incompleteness were the same for both indicators, namely, high family income, high parity, low number of prenatal consultations, no application of Tdap during pregnancy, no breastfeeding at age 12 months and no use of the public health care services for vaccination.

Our analyses revealed that children from the better-off families has greater risk of incompleteness when compared with the poorest quintile of family income. Lower coverage in the richest was previously reported in Brazil [14, 17]. Barata et al., assessed vaccine coverage of children born in 2008 in 27 state capitals and in Brasilia and showed that in eight cities coverage was lower among children belonging to wealthier socioeconomic classes [17]. In a study carried out in four cities of São Paulo state, Moraes et al. also found a negative relation between socioeconomic stratum and immunization [14]. These findings suggest that vaccine hesitancy, already observed among well-off families in high-income settings [25], may have appeared in our country. To address this scenario, strategies to spread information based on scientific evidence are needed [26].

Additionally, low socioeconomic status has been found as one of the main factors associated with incomplete vaccination in Brazil [10, 12, 27] and around the globe [28]. It is worth mentioning that, in our analyses, higher coverage of incomplete immunization was also found among the poorest when compared with the three middle quintiles of family income **(**Additional file 3**)**. This is important to consider, as these children are at higher risk of develop infectious diseases due to their poorer living conditions.

It is well described in the literature the association between higher number of children in the family and incomplete immunization [12, 17, 28–30]. In line with the published evidence, we found that the risk of incomplete vaccination increases with higher parity. This finding may reflect the socioeconomic status -poorer families tend to be more numerous-, but also the difficulty of mothers with many children to get to the health care services. These mothers may have less time to commit to the care of an individual child and may need to organize the childcare for other siblings, thus making the vaccination not a priority.

Consistent with a study carried out in Maranhão state [12], children of mothers who did not attend prenatal care as suggested by the Ministry of Health (at least six visits) were more likely to be incompletely vaccinated. Also, we found that risk of incompleteness was higher for those children whose mothers did not get the Tdap vaccine during pregnancy. These results may indicate that pregnant women who follow the recommendations in terms of prenatal care (number of consultations and administration of mandatory vaccines for pregnancy) show a greater adherence to health practices and services, including their child’s vaccination. Along similar lines, the risk of incomplete vaccination was higher for those children whose mothers did not breastfed them by age 12 months. We hypothesized that mothers who practice prolonged breastfeeding, may have access to evidence-based information about health in general, thus, trusting on vaccination [24, 31].

In line with our results, prior studies in Brazil showed that the involvement of the public health service in vaccination improved coverage [32], emphasizing the need of adequate funding to provide services of good quality and achieve immunization’s goals [32, 33].

We acknowledge some limitations of this study. First, 10% of the information on immunization relied on maternal recall. Previous studies that compared parental recall and card-based data on children’s immunization have shown that can exist either underestimations or overestimations, but in general, the recall provide accurate information [34, 35]. Second, we possibly overestimated vaccine coverage in the first year of life because the information was collected at the age of 2 years. Nevertheless, this is unlikely to have affected the association analyses. Third, we had no information regarding immunization for 8% of the sample. However, these missing values were not related with any of the covariates, thus reducing the possibility of bias. Lastly, another limitation was the lack of data related to access to health care facilities and availability of vaccines, and parental knowledge and attitudes regarding vaccination, necessary to better understand the whole picture of barriers that affect immunization coverage.

Strengths of our study include the large population-based cohort which represents around 99% of all births in our city and the low attrition rate (4.8% at the 2-year follow-up), which minimize the possibility of selection bias. We believe our findings are generalizable to all children of the city and other cities with similar characteristics. Moreover, our analyses are based on primary data, collected by trained interviewers and standardized techniques, thus providing a more accurate estimate of the coverage indicators when compared with data collected from computerized systems or health facilities.

## Conclusion

Adequate coverage, according to the parameters established for each vaccine, is essential to reducing child morbidity and mortality [36]. Overall, our results showed that childhood immunization coverage in the 2015 Birth Cohort are inadequate. This situation may put many children at risk of potentially severe but preventable diseases, making necessary the development and implementation of policies to tackle incomplete immunization among children. The fact that both extremes of socioeconomic strata had lower coverage underlines the need of new strategies to improve the access and the quality of immunization services. We believe that efforts to increase vaccination should also address pregnant women, for example, obstetricians and family doctors can be used to promote vaccination during prenatal visits [37]. Finally, we propose to strengthen the promotion of immunization through the dissemination of high-quality information, especially between well-off families.

## Supplementary information

**Additional file 1.** 2015 vaccination schedule for the first year of life in Brazil.

**Additional file 2.** Characteristics of the sample. 2015 Pelotas Birth Cohort.

**Additional file 3.** Basic and expanded incomplete immunization according to independent variables. 2015 Pelotas Birth Cohorts.

## Data Availability

Due to confidentiality restrictions related to the ethics approval for this study, no identifying information about participants may be released. Dataset without identification used during the current study are available from the corresponding author on reasonable request.

## Abbreviations

BCG: Bacille Calmette-Guérin
Tdap: Tetanus toxoid, reduced difteria toxoid and acellular pertussis
