# Supplementary material for: Vaccine coverage within the first year of life and associated factors with incomplete immunization in a Brazilian birth cohort
Source: Arch Public Health. 2020 Apr 8;78:21. doi: 10.1186/s13690-020-00403-4 (PMC7140489; doi:10.1186/s13690-020-00403-4)
Supplement: Supplementary file 1 — Additional file 1. 2015 vaccination schedule for the first year of life in Brazil. [file 13690_2020_403_MOESM1_ESM.docx]

**Additional file 1. 2015 vaccination schedule for the first year of life in Brazil.**

| **Age (months)** | **Vaccines** | | | | | | | | |
| --- | --- | --- | --- | --- | --- | --- | --- | --- | --- |
|  | **BCG^a^** | **Poliovirus (OPV/ IPV^b^)** | **Pentavalent^c^** | **MMR^d^** | **Hepatitis B** | **10-valent pneumococcal conjugate** | **C meningococcal** | **Rotavirus** | **Hepatitis A** |
| **0 (birth)** | **x** |  |  |  | **x** |  |  |  |  |
| **1** |  |  |  |  |  |  |  |  |  |
| **2** |  | **x** | **x** |  |  | **x** |  | **x** |  |
| **3** |  |  |  |  |  |  | **x** |  |  |
| **4** |  | **x** | **x** |  |  | **x** |  | **x** |  |
| **5** |  |  |  |  |  |  | **x** |  |  |
| **6** |  | **x** | **x** |  |  | **x** |  |  |  |
| **7** |  |  |  |  |  |  |  |  |  |
| **8** |  |  |  |  |  |  |  |  |  |
| **9** |  |  |  |  |  |  |  |  |  |
| **10** |  |  |  |  |  |  |  |  |  |
| **11** |  |  |  |  |  |  |  |  |  |
| **12** |  |  |  | **x** |  |  |  |  | **x** |
| **x** indicates one dose  **^a^**BCG: bacilleCalmette-Guérin  **^b^**OPV: oral polio vaccine / IPV: inactivated polio vaccine  **^c^**Pentavalent: diphtheria–tetanus–pertussis plus Haemophilus influenza type b and hepatitis B  **^d^**MMR: measles-mumps-rubella | | | | | | | | | |

Adapted from the National Schedule, Ministry of Health, Brazil
