# Supplementary material for: Vaccine coverage within the first year of life and associated factors with incomplete immunization in a Brazilian birth cohort
Source: Arch Public Health. 2020 Apr 8;78:21. doi: 10.1186/s13690-020-00403-4 (PMC7140489; doi:10.1186/s13690-020-00403-4)
Supplement: Supplementary file 2 — Additional file 2. Characteristics of the sample. 2015 Pelotas Birth Cohort. [file 13690_2020_403_MOESM2_ESM.docx]

| **Additional file 2. Characteristics of the sample. 2015 Pelotas Birth Cohort** | | |
| --- | --- | --- |
|  | **N** | **%** |
| **Maternal education (years)** |  |  |
| 0 to 4 | 356 | 8.9 |
| 5 to 8 | 1036 | 25.8 |
| 9 to 11 | 1385 | 34.5 |
| 12 or more | 1235 | 30.8 |
| **Maternal age (years)** |  |  |
| < 20 | 578 | 14.4 |
| 20 - 35 | 2985 | 74.4 |
| > 35 | 451 | 11.2 |
| **Maternal skin color** |  |  |
| White | 2875 | 71.7 |
| Brown | 524 | 13.1 |
| Black | 611 | 15.2 |
| **Parity** |  |  |
| 1 child | 2012 | 50.1 |
| 2 children | 1238 | 30.9 |
| 3 children or more | 762 | 19.0 |
| **Number of prenatal consultations** |  |  |
| 0 to 5 | 518 | 13.2 |
| 6 or more | 3410 | 86.8 |
| **Tdap^a^ vaccine during pregnancy** |  |  |
| No | 1603 | 42.3 |
| Yes | 2185 | 57.7 |
| **Breastfedding status at 12 months** |  |  |
| No | 2290 | 58.7 |
| Yes | 1614 | 41.3 |
| **Use of public health care services** |  |  |
| No | 423 | 10.5 |
| Yes | 3588 | 89.5 |
| **^a^** Tdap: tetanus toxoid, reduced difteria toxoid and acellular pertussis  Highest number of missing data: 226 (Tdap vaccine during pregnancy) and 86 (number of prenatal consultations) | | |
