# Supplementary material for: Vaccine coverage within the first year of life and associated factors with incomplete immunization in a Brazilian birth cohort
Source: Arch Public Health. 2020 Apr 8;78:21. doi: 10.1186/s13690-020-00403-4 (PMC7140489; doi:10.1186/s13690-020-00403-4)
Supplement: Supplementary file 3 — Additional file 3. Basic and expanded incomplete immunization according to independent variables. 2015 Pelotas Birth Cohorts. [file 13690_2020_403_MOESM3_ESM.docx]

| **Additional file 3. Basic and expanded incomplete immunization according to independent variables. 2015 Pelotas Birth Cohorts.** | | | | |
| --- | --- | --- | --- | --- |
|  | Basic^a^ | | Expanded^b^ | |
|  | % | 95% IC | % | 95% IC |
| **Maternal education (years)** | |  |  |  |
| 0 to 4 | 27.0 | 22.6; 31.8 | 37.9 | 33.0; 43.1 |
| 5 to 8 | 22.0 | 19.5; 24.6 | 35.4 | 32.6; 38.4 |
| 9 to 11 | 19.0 | 17.0; 21.1 | 29.8 | 27.4; 32.2 |
| 12 or more | 26.6 | 24.2; 29.2 | 36.1 | 33.5; 38.8 |
| **Family income (quintiles)** | |  |  |  |
| Q1 (poorest) | 24.1 | 21.2; 27.2 | 37.1 | 34.1; 40.8 |
| Q2 | 20.6 | 17.9; 23.5 | 31.9 | 28.8; 35.2 |
| Q3 | 19.1 | 16.5; 21.9 | 29.2 | 26.1; 32.4 |
| Q4 | 19.6 | 17.0; 22.5 | 30.1 | 27.0; 33.3 |
| Q5 (richest) | 31.0 | 27.9; 34.3 | 41.2 | 37.8; 44.6 |
| **Maternal age (years)** |  |  |  |  |
| < 20 | 20.1 | 17.0; 23.5 | 31.8 | 28.2; 35.7 |
| 20 - 35 | 22.7 | 21.2; 24.2 | 33.9 | 32.2; 35.6 |
| > 35 | 27.7 | 23.8; 32.0 | 36.8 | 32.5; 41.4 |
| **Maternal skin color** |  |  |  |  |
| White | 23.2 | 21.7; 24.7 | 33.7 | 32.0; 35.4 |
| Brown | 21.6 | 18.3; 25.3 | 34.7 | 30.8; 38.9 |
| Black | 22.4 | 19.3; 25.9 | 34.2 | 30.5; 38.1 |
| **Parity** |  |  |  |  |
| 1 child | 18.9 | 17.2; 20.7 | 29.1 | 27.2; 31.2 |
| 2 children | 24.7 | 22.4; 27.2 | 35.3 | 32.7; 38.0 |
| 3 children or more | 30.2 | 27.0; 33.5 | 44.2 | 40.7; 47.8 |
| **Number of prenatal consultations** | | |  |  |
| 0 to 5 | 31.7 | 27.8; 35.8 | 44.8 | 40.6; 49.1 |
| 6 or more | 21.0 | 19.7; 22.4 | 31.6 | 30.1; 33.2 |
| **Tdap^c^ vaccine during pregnancy** | |  |  |  |
| No | 24.9 | 22.8; 27.1 | 37.4 | 35.0; 39.8 |
| Yes | 20.9 | 19.3; 22.7 | 30.6 | 28.7; 32.6 |
| **Breastfedding status at 12 months** | | |  |  |
| No | 22.6 | 20.6; 24.0 | 34.6 | 32.7; 36.6 |
| Yes | 18.6 | 16.8; 20.6 | 28.6 | 26.5; 30.9 |
| **Use of public health care services** | | |  |  |
| No | 30.5 | 26.3; 35.1 | 41.1 | 36.5; 45.9 |
| Yes | 21.9 | 20.6; 23.3 | 33.0 | 31.5; 34.6 |
| **^a^**Basic incomplete immunization was defined as have not reached one dose of BCG, one dose of MMR, three doses of polio vaccine, and three doses of pentavalent vaccine. | | | | |
| **^b^**Expanded incomplete immunization was defined as have not reached the complete scheduled vaccine doses of national immunization program by age 12 months ( FIC basic + one dose of hepatitis B, one dose of hepatitis A, three doses of 10-valent pneumococcal, two doses of rotavirus and two doses of C meningococcal) | | | | |
| **^c^** Tdap: tetanus toxoid, reduced difteria toxoid and acellular pertussis | | | | |
